# Epithelial CD74 regulates intestinal barrier homeostasis

**DOI:** 10.3389/fimmu.2026.1856250

**Published:** 2026-10-30

**Authors:** Masako Kohyama, Koichi Iwatani, Sakiko Tomita, Takayoshi Ito, Hisako Kayama, Daisuke Motooka, Hisashi Arase, Kiyoshi Takeda, Wataru Ise

**Affiliations:** 1 Regulation of Host Defense Team, Division of Microbiology and Immunology, Center for Infectious Disease Education and Research, Osaka University, Suita, Osaka, Japan; 2 Laboratory of Immune Regulation, Department of Microbiology and Immunology, Graduate School of Medicine, University of Osaka, Suita, Osaka, Japan; 3 World Premier International Research Center Initiative (WIP) Immunology Frontier Research Center, University of Osaka, Suita, Osaka, Japan; 4 Institute for Advanced Co-Creation Studies, University of Osaka, Suita, Osaka, Japan; 5 Integrated Frontier Research for Medical Science Division, Institute for Open and Transdisciplinary Research Initiatives (OTRI), Osaka University, Suita, Osaka, Japan; 6 Center for Infectious Disease Education and Research, University of Osaka, Suita, Osaka, Japan; 7 Center for Advanced Modalities and Drug Delivery System (DDS), Osaka University, Osaka, Suita, Japan

**Keywords:** CD74, epithelial cells, homeostasis, intestine, mitochondria

## Abstract

CD74, originally identified as the invariant chain of MHC class II, also functions as a receptor for macrophage migration inhibitory factor (MIF). Although CD74 is expressed in various non-lymphoid cells, including epithelial cells across multiple organs, the regulation and functional significance of its expression in these tissues remain poorly understood. Here, we show that CD74 is constitutively expressed in small intestinal epithelial cells, particularly within stem and transit-amplifying compartments, and that its expression is influenced by microbiota and IFN-γ signaling. Conditional deletion of CD74 in IECs (CD74^ΔIEC^ mice) resulted in increased intestinal permeability under steady-state conditions, which was consistent with reduced expression of the tight junction protein ZO-1. While the numbers of intestinal stem cells and Paneth cells remained unchanged, CD74-deficient mice also exhibited elongation of the small intestine and enhanced crypt cell proliferation. Single-cell RNA sequencing revealed enrichment of mitochondrial pathways in CD74-deficient IECs. Consistent with these findings, CD74-deficient IECs exhibited increased mitochondrial content and altered mitochondrial membrane potential. Organoid cultures further showed accelerated growth and increased proliferation in the absence of CD74. Together, these findings suggest that CD74 contributes to intestinal epithelial homeostasis by limiting epithelial proliferation and supporting tight junction integrity, thereby helping to maintain barrier function under steady-state conditions.

## Introduction

Increased intestinal permeability, often referred to as “leaky gut,” reflects disruption of the epithelial barrier that permits luminal antigens and microbial products to access the systemic compartment. Barrier dysfunction has been implicated in gastrointestinal disorders such as celiac disease, Crohn’s disease, and irritable bowel syndrome, as well as in autoimmune and allergic diseases (1, 2). Maintenance of epithelial homeostasis requires a tightly regulated balance of proliferation, differentiation, migration, and polarity (3). Perturbation of this balance compromises barrier integrity and predisposes to pathology (4).

CD74 is a type II transmembrane protein consisting of a short cytoplasmic tail, a transmembrane domain, and a luminal region (5). In professional antigen-presenting cells, CD74 functions as the invariant chain of MHC class II molecules, acting as a chaperone during antigen presentation (6–8). Beyond this canonical role, CD74 also serves as a cell surface receptor independent of MHC class II (9). Notably, CD74 was identified as a high-affinity receptor for macrophage migration inhibitory factor (MIF), a pleiotropic cytokine implicated in infectious, autoimmune, cardiovascular, and malignant diseases (10–13). MIF is secreted by both immune and non-immune cells, including epithelial cells (12). Previous studies have reported that loss of MIF disrupts tight and adherents junctions in the colon, leading to increased intestinal permeability (12), suggesting a role for MIF-dependent signaling in the regulation of intestinal homeostasis. These effects may be mediated either directly through epithelial CD74 signaling or indirectly via immune cell–dependent mechanisms. CD74 is expressed in epithelial cells across multiple organs, including the intestine (14–17). However, most studies have focused on its role in inflammatory contexts. Consequently, the physiological significance of epithelial CD74 in maintaining intestinal homeostasis under steady-state conditions remains poorly understood.

In this study, we investigated the epithelial-intrinsic function of CD74 in the small intestine under homeostatic conditions. We demonstrate that CD74 expression in intestinal epithelial cells is influenced by microbiota and IFN-γ signaling. Using mice with IEC-specific deletion of CD74, we show that loss of CD74 results in increased intestinal permeability accompanied by enhanced crypt proliferation and alterations in mitochondrial content and membrane potential. Our findings suggest that CD74 may function as a homeostatic regulator coordinating epithelial metabolic state, proliferative activity, and barrier integrity.

## Materials and method

### Mice

C57BL/6JJcl mice were purchased from CLEA Japan (Tokyo, Japan). CD74^fl/fl^ mice were generated as previously described (18, 19). Ifngr^fl/fl^ and Villin-Cre mice were obtained from The Jackson Laboratory (Bar Harbor, ME, USA). CD74ΔIEC mice were generated by crossing CD74^fl/fl^ mice with Villin-Cre transgenic mice. Littermate CD74^fl/fl^ mice lacking Cre were used as wild-type controls unless otherwise indicated. Both male and female mice were used at 8–12 weeks of age. Mice were housed under specific pathogen-free conditions at Osaka University. Experiments were performed using age- and sex-matched animals. All animal experiments were approved by the Institutional Animal Care and Use Committee of Osaka University.

### Reagents

FITC–dextran (FD4), DNase I (DN25), and BSA (A4503) were from Sigma-Aldrich (St. Louis, MO, USA). TrypLE Express (12604–021), and Dispase II (17105–041), GlutaMAX (35050061), HEPES (15630080), B27-Serum-free Supplement (17504044), N-2 Supplement (17502048), SYTOX Blue Dead cell stain (S34857) were obtained from Thermo Fisher Scientific (Waltham, MA, USA). Hanks’ balanced salt solution (HBSS) (17460–15), Dulbecco’s modified Eagle’s medium (DMEM)/Ham’s F-12 (08460–95), DMEM high-glucose (08458–16), 4’,6-diamidino-2-phenylindole dihydrochloride (DAPI: 11034-56), and sodium azide (31233–42) were purchased from Nacalai Tesque (Kyoto, Japan). Murin Noggin (250–38), anti-ZO-1 (21773-AP) were from PeproTech (Rocky Hill, NJ, USA). Murin R-spondin1(715-RS-050) was from R&D (Minneapolis, MN, USA). Anti-B220 (553088), anti-CD11b (562950), anti-CD11c (562949), anti-CD44 (5600567), anti-CD5 (740444) were purchased from BD Biosciences (Franklin Lakes, NJ, USA). Anti-Ki67 (652402), anti-CD4 (100438), anti-CD45.1 (110708), anti-B220 (103222, 103247), anti-CD62L (104437), anti-GL7 (144614), anti-CD38 (102717), anti-CD74 (151006), anti-MHC II (107614), anti-CD24 (138503), anti-CD31 (102417), andi-CD326 (118218), anti-BrdU (229810), anti-actin (622101),were purchased from BioLegend (San Diego, CA, USA). Anti-CD69 (17–0691) and SuperScript III (18080–051) were purchased from Invitrogen (Waltham, MA, USA). Anti-IgA was purchased from SouthernBiotech (Brimingham, LA, USA). Anti-Lysozyme C-1/2 (60487S) was from Cell signaling Technology (Danvers, MA, USA).

### Isolation of intestinal epithelial cells for flow cytometry

Small intestines were excised, flushed with ice-cold PBS, and opened longitudinally. Tissues were cut into 1–2 cm pieces and washed extensively with PBS. To detach epithelial cells, fragments were incubated in PBS containing 5 mM EDTA at 37 °C for 30 min with gentle agitation (200 rpm). Released cells were filtered through a 100-µm cell strainer, washed, and centrifuged at 300 × g for 5 min at 4 °C. Pellets were digested in TrypLE Express for 20 min at 37 °C with gentle pipetting. Digestion was washed with FACS buffer (PBS, 2% FBS, 2 mM EDTA), and suspensions were sequentially filtered through 70-µm and 40-µm strainers.

### Oral antibiotic treatment and fecal microbiota transplantation

For microbiota depletion, an antibiotic cocktail consisting of vancomycin, metronidazole, cefoxitin and gentamicin (final concentrations 1 mg/ml each), or single vancomycin were added to the drinking water for 3weeks before analysis (20). The control mice received control water without antibiotics. For fecal microbiota transplantation (FMT), fresh fecal pellets collected from untreated donor mice were suspended in sterile PBS and administered orally once daily for 7 consecutive days following antibiotic withdrawal. Mice were analyzed 7 days after completion of FMT.

### Flow cytometric analysis

For flow cytometry, cells were pre-incubated with anti-CD16/32 and stained with antibodies for 30 min at 4 °C. Dead cells were excluded with SYTOX Blue. Data were acquired on BD FACSAria III (BD Biosciences) and analyzed using FlowJo software (Tree Star).

### Organoid culture

Intestinal crypts were isolated by incubating small intestine fragments in 10 mM EDTA for 15 min at room temperature with gentle rotation. Crypts were released by vigorous shaking, filtered through a 70-µm strainer, and washed with PBS. Crypts were embedded in Matrigel and cultured in Advanced DMEM/F12 supplemented with 1× GlutaMAX, 10 mM HEPES, 1× B27, 1× N2, 100 ng/mL murine Noggin, and 500 ng/mL R-spondin1. Medium was changed every 2–3 days. For MIF stimulation experiments, recombinant mouse MIF (100 ng/mL) was added to the culture medium at day 2 and maintained until analysis. For inhibition experiments, ISO-1 was used at 20 µM. Bright-field images were analyzed using Fiji (ImageJ), and organoid area was manually traced using the freehand selection tool.

### Histology and immunostaining

Small intestinal tissues were fixed in 4% paraformaldehyde (PFA) overnight at 4 °C. For paraffin sections, tissues were dehydrated, embedded, and cut into 5 µm sections. For cryosections, tissues were equilibrated in 30% sucrose, embedded in OCT compound (Sakura Finetek), frozen in liquid nitrogen, and sectioned at 7–10 µm (Leica CM3050 S). H&E, periodic acid–Schiff (PAS), and alkaline phosphatase (ALP) staining were performed using standard protocols. For immunofluorescence, sections were permeabilized with 0.1% Triton X-100 in PBS and blocked in 5% normal goat serum for 1 h at room temperature. Sections were incubated with primary antibodies (anti-Ki67, anti-ZO-1, anti-OLFM4, anti-Lysozyme) overnight at 4 °C, followed by Alexa Fluor–conjugated secondary antibodies (1:500) for 1 h at room temperature. Nuclei were counterstained with DAPI (1 µg/mL). For immunohistochemistry, endogenous peroxidase was quenched with 0.3% H_2_O_2_ in methanol. After blocking, sections were incubated with primary antibodies overnight at 4 °C, followed by HRP-conjugated secondary antibodies (1:500; Vector Laboratories). Signals were visualized with DAB substrate (Vector Laboratories), counterstained with hematoxylin, dehydrated, and mounted with Permount. Images were acquired with a BZ-X1000 microscope (Keyence).

### Intestinal permeability assay

Mice were fasted for 4 h with free access to water prior to the assay. FITC–dextran (4 kDa; 50 mg per 100 g body weight) was administered by oral gavage. After 4 h, blood was collected and serum fluorescence intensity was measured using a microplate reader (excitation 485 nm, emission 528 nm). FITC–dextran concentration was calculated using a standard curve generated with serial dilutions of FITC–dextran in mouse serum.

### Radiation injury model

Mice were subjected to total body irradiation (9.5 Gy, single dose) using an X-ray irradiator. Body weight was monitored daily for 14 days. Survival and clinical signs were recorded. Blood samples were collected at indicated time points for 16S rRNA quantification. Histological injury was evaluated in hematoxylin and eosin (H&E)-stained sections by a blinded observer. Intestinal inflammation and tissue damage were scored separately. Inflammation was graded on a scale of 0–3 as follows: 0, no inflammatory infiltrate; 1, mild inflammatory cell infiltration; 2, moderate inflammatory cell infiltration readily detectable within the lamina propria; and 3, severe inflammatory cell infiltration involving most of the lamina propria. Tissue damage was graded on a scale of 0–3 as follows: 0, normal tissue architecture; 1, mild epithelial injury or focal villus shortening; 2, moderate villus shortening and epithelial disruption; and 3, severe epithelial destruction, erosion, or ulceration. The total histological injury score was calculated as the sum of the inflammation and tissue damage scores.

### RNA extraction and qPCR

Total RNA was isolated using TRIzol reagent and reverse-transcribed with SuperScript III. qPCR was performed using Power SYBR Green PCR Master Mix on a QuantStudio 3 system (Applied Biosystems).

### Single-cell RNA sequencing

Single-cell suspensions of intestinal epithelial cells were prepared as described above. Libraries were generated with the Chromium Next GEM Single Cell 3’ Kit v3.1 (10x Genomics) following the manufacturer’s protocol. In one of the two datasets, samples were multiplexed using TotalSeq™-A hashtag oligonucleotide-conjugated antibodies (Biolegend), and sample identities were assigned by demultiplexing hashtag counts. Sequencing was performed on an Illumina NovaSeq 6000, and raw data were processed using Cell Ranger (10x Genomics). After initial processing, the two scRNA-seq datasets were merged into a single object for downstream analysis. Quality control filtering, dimensionality reduction, cell clustering and visualization were carried out with Python (v.3.10.11) package Scanpy (v.1.10.2) (21). In brief, low-quality cells (defined as cells with fewer than 200 genes) and genes (defined as genes expressed in fewer than 3 cells) were removed. Putative doublets were also removed. Cells were filtered based on the number of genes by counts (less than 7000) and the percentage of mitochondrial reads (less than 20%). After data normalization and detection of the highly variable genes, dimensionality reduction was performed using principal component analysis. Dimensionality reduction and batch correction were carried out, and then cells were clustered using the Leiden algorithm. These Leiden-based cell clusters were manually annotated using well-known cell-type specific genes. A dot plot and violin plots were visualized using Scanpy function. Differentially expressed gene analysis were performed by Scanpy function. Pathway activity inference was performed using decoupler (22) based on Gene Ontology-derived gene sets.

### Statistical analysis

Statistical analyses were performed with GraphPad Prism v8.4.3. Two-tailed unpaired Student’s t-tests or one-way ANOVA with Tukey’s *post hoc* test were used as appropriate. P values <0.05 were considered statistically significant.

## Results

### CD74 is differentially expressed within intestinal epithelia and induced by the microbiota

To clarify the role of CD74 in the intestinal tract, we first analyzed its expression in intestinal epithelial cells (IECs) under homeostatic conditions in wild-type C57BL/6 mice by immunohistochemistry. CD74 expression was barely detectable in the colon but was evident in the small intestine, with higher levels in the ileum than in other regions (**Figure 1A**). This pattern is consistent with a recent report on MHC class II expression along the intestinal tract (23). We next examined CD74 expression on CD45^-^EpCAM^+^ IECs by flow cytometry. Intracellular CD74 was similarly detected in almost all IECs and splenic B cells. By contrast, surface CD74 expression differed between the two cell types: while splenic B cells exhibited uniformly low levels, a substantial fraction of IECs (50–70%) showed high surface expression (**Figure 1B**). Collectively, these findings demonstrate that CD74 is abundantly expressed on the epithelial cell surface, suggesting that it may function as a signaling receptor in IECs.

**Figure 1:**
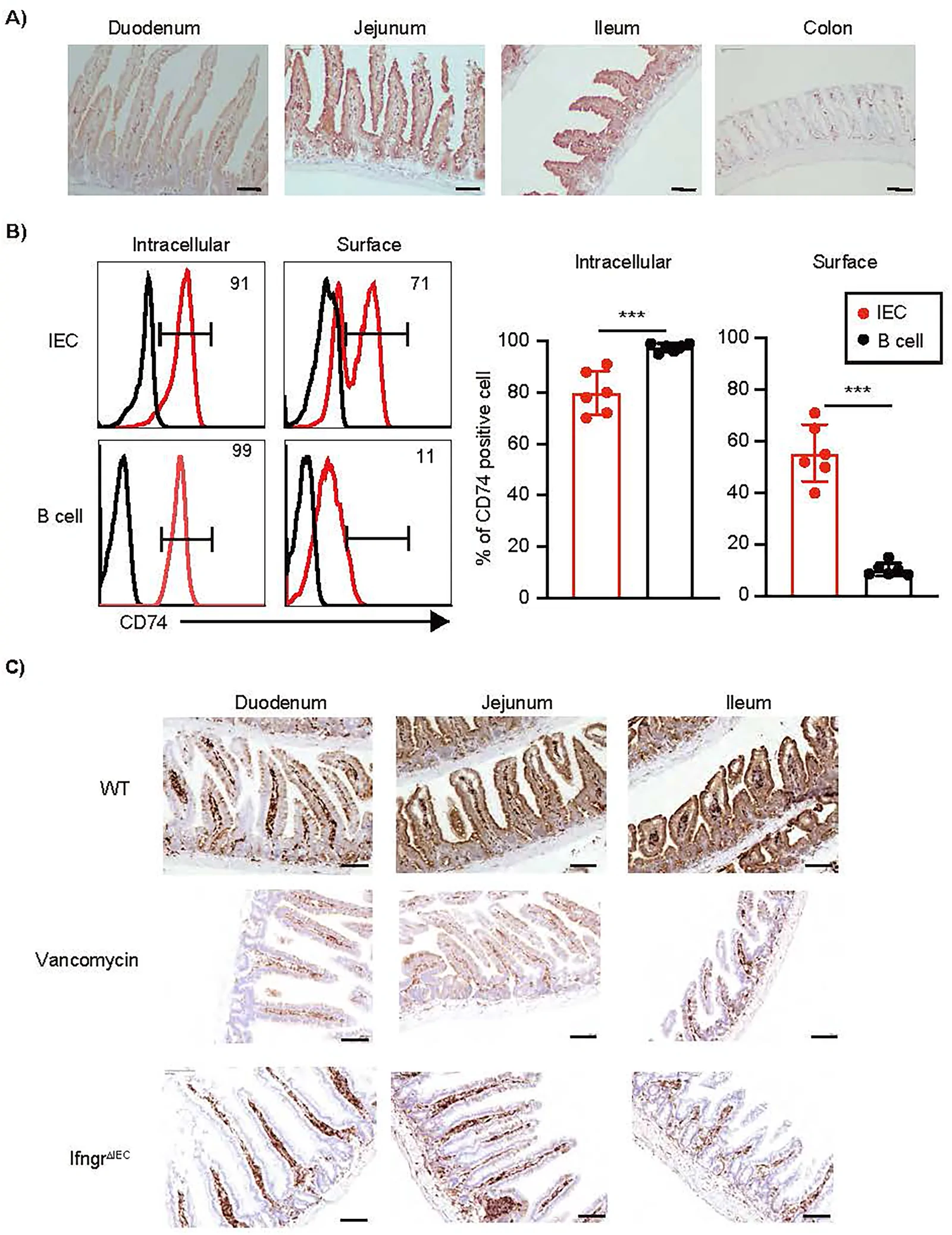
CD74 is expressed along the murine intestinal epithelium and regulated by the microbiota. **(A)** Representative immunohistochemistry of CD74 in the duodenum, jejunum, ileum, and colon of naïve WT mice. Scale bars, 100 μm. **(B)** Flow cytometric analysis of surface and intracellular CD74 expression on intestinal epithelial cells (IECs) from the distal small intestine and on splenic B cells from naïve mice. Representative plots and quantification of the percentage of surface and intracellular CD74-positive cells are shown (n=6). **(C)** Representative staining of CD74 expression in the duodenum, jejunum, ileum of vancomycin-treated, Ifngr^ΔIEC^ and WT control mice. Scale bars, 100 μm.

Previous studies have shown that germ-free mice or vancomycin-treated mice exhibit reduced MHC class II expression on IECs (23, 24). To test whether CD74 expression on IECs is microbiota-dependent, we first assessed CD74 levels in antibiotic-treated mice. As expected, CD74 expression on IECs was markedly reduced in vancomycin-treated mice, whereas expression on lamina propria cells was unaffected (**Figure 1C**; **Supplementary Figures 1A, B**). Notably, low-level CD74 expression remained detectable after vancomycin treatment. To more broadly deplete the intestinal microbiota, mice were treated with a cocktail of four antibiotics (cefoxitin, gentamicin, metronidazole, and vancomycin). This treatment resulted in a comparable reduction of epithelial CD74 expression, with only minimal expression remaining (**Supplementary Figures 1A, B**). Importantly, fecal microbiota transfer restored epithelial CD74 expression, indicating that microbial stimulation contributes to the maintenance of CD74 expression on IECs (**Supplementary Figure 1C**).

Next, we examined CD74 expression in IEC-specific IFN-γ receptor-deficient (Ifngr^ΔIEC^) mice. In Ifngr^ΔIEC^ mice, CD74 expression was markedly diminished in epithelial cells, whereas its expression in lamina propria lymphocytes was unchanged (**Figure 1C**). To determine whether IFN-γ directly induces CD74 expression in intestinal epithelial cells, primary intestinal organoids established from WT mice were stimulated with recombinant IFN-γ. IFN-γ increased surface CD74 expression on epithelial cells (**Supplementary Figure 2A**), supporting a direct role for IFN-γ in the induction of epithelial CD74 expression. Together, these results indicate that steady-state expression of CD74 on IECs is regulated by the intestinal microbiota and is driven largely by IFN-γ signaling. Nevertheless, the persistence of CD74 expression in the absence of IFN-γ signaling suggests that additional IFN-γ-independent factors contribute to the regulation of epithelial CD74 expression.

### Increased intestinal permeability in CD74 deleted mice

To assess the role of CD74 on IECs under homeostatic conditions, we examined a CD74 conditional knock-out model. We crossed CD74^fl/fl^ mice (18, 19) with Villin-cre mice, generating a mouse model of specific CD74 deficiency in IECs (CD74^ΔIEC^). CD74 was specifically deleted in the intestinal epithelium; notably, its expression was preserved in LPL (**Figure 2A**) as well as in other immune subsets such as splenic B cells as confirmed by flow cytometry (**Supplementary Figure 3A**). Because CD74 functions as a chaperone for MHC II, global deletion of CD74 results in defective CD4^+^ T cell differentiation due to impaired MHC II expression (25). In contrast, in CD74^ΔIEC^ mice, MHC II expression was maintained (**Supplementary Figures 3B–D**). As a result, CD4^+^ T cell differentiation proceeds normally in this model (**Supplementary Figure 5A**). Consequently, CD74^ΔIEC^ mice offer a distinct advantage over systemic knockouts by allowing for the precise analysis of epithelial-specific roles without the interference of systemic phenotypes (25). Previous studies have shown that MIF activates ERK signaling through CD74 (10–13). We therefore asked whether the MIF-CD74 axis similarly mediates ERK activation in intestinal epithelial cells. In Caco-2 cells, MIF-induced ERK phosphorylation was reduced by the CD74-blocking antibody LN2 (**Supplementary Figure 2B**). Consistent with these findings, MIF-induced ERK phosphorylation was readily detected in WT intestinal organoids but was absent in organoids derived from CD74^ΔIEC^ mice (**Supplementary Figure 2C**), supporting a role for epithelial CD74 in MIF-induced ERK activation.

**Figure 2:**
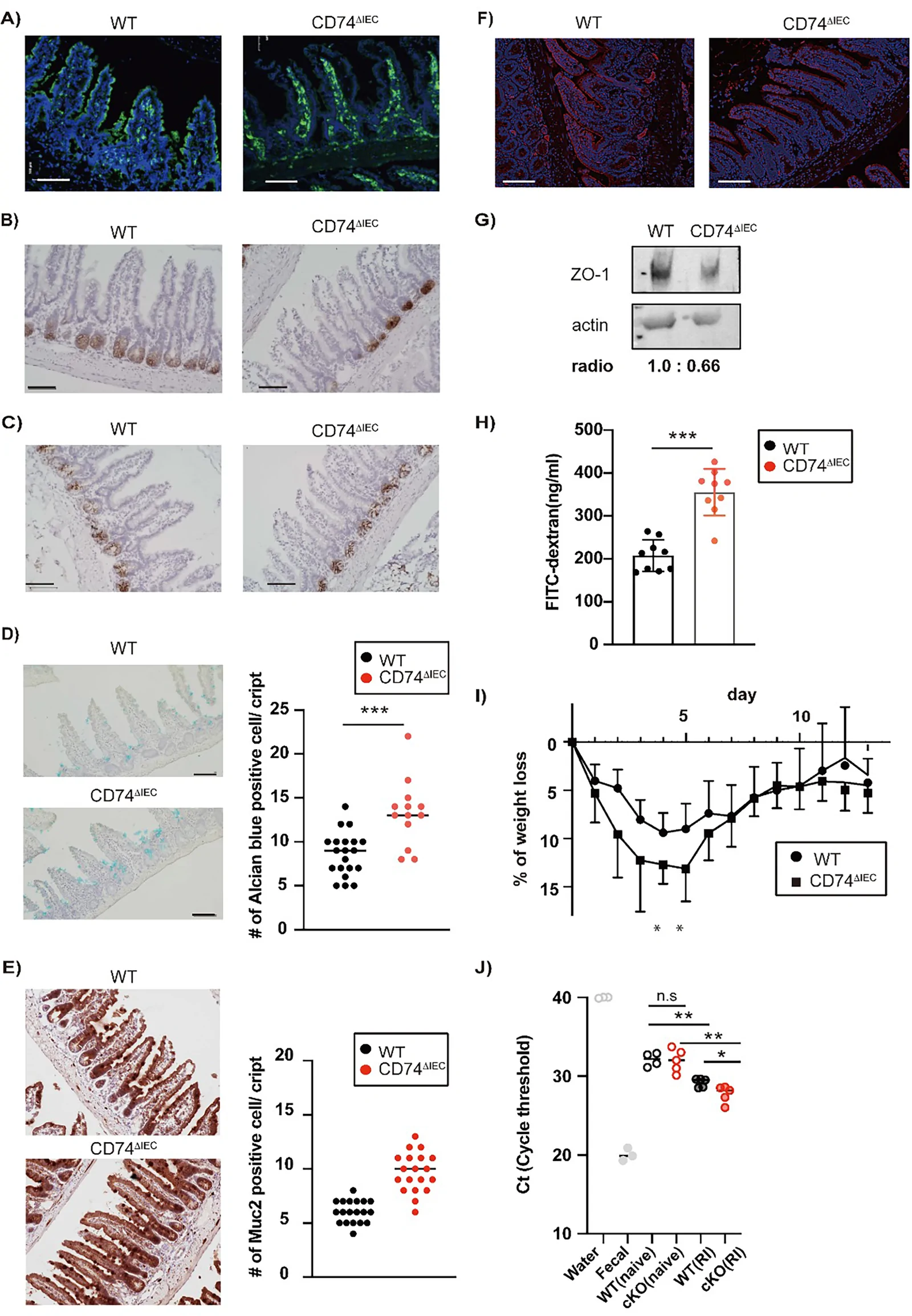
Intestinal CD74 deletion increases gut permeability and susceptibility to injury. **(A)** Representative immunostaining of CD74 expression in distal small intestine of CD74^ΔIEC^ and WT mice. Scale bars, 100 µ. **(B, C)** Representative histograms for lysozyme (LYZ) **(B)** and OLFM4 **(C)** of distal small intestine from CD74^ΔIEC^ mice and WT littermate controls. Scale bars, 100 µm. **(D)** Alcian blue staining and quantification of Alcian blue positive cells per crypt of distal small intestine from CD74^ΔIEC^ mice and control WT littermate controls. Scale bars, 100 µm. ***p<0.001 **(E)** Muc2 staining and quantification of Muc2 positive cells per crypt of distal small intestine from CD74^ΔIEC^ mice and WT littermate controls. Scale bars, 100 µm. ***p<0.001 **(F)** Representative immunostaining of ZO-1 in the distal small intestine of CD74^ΔIEC^ mice and WT littermate controls. Scale bars, 100 μm. **(G)** Representative immunoblot of ZO-1 protein expression in the distal small intestine of CD74^ΔIEC^ mice and WT littermate controls. Relative ZO-1 protein expression normalized to β-actin is shown on the right (WT = 1). Quantification represents n=2 biologically independent mice per group. Similar results were obtained in two independent experiments. **(H)** Serum FITC–dextran concentration after oral gavage in CD74^ΔIEC^ mice and WT littermate controls. Representative data from two independent experiments is shown. ***p<0.001. **(I)** Body weight change (% of baseline) following total body irradiation (TBI). Data are pooled from two independent experiments (n = 5–6 per group). *p<0.05. **(J)** Quantitative PCR for bacterial 16S rRNA gene in serum. Ct values are shown, with water and SPF mouse fecal DNA as negative and positive controls, respectively, confirming bacterial translocation in irradiated CD74-deficient mice. *p<0.05, **p<0.01.

Upon deletion of CD74 in the intestinal epithelium, no overt pathological abnormalities were observed under steady-state conditions. The gross morphology and crypt–villus architecture of both the small intestine were not altered (**Supplementary Figure 4**). Staining for OLFM4 and LYZ showed comparable expression patterns to those observed in wild-type controls (**Figures 2B, C**), indicating preserved stem cell and Paneth cell compartments. Furthermore, evaluation of immune cell activation and humoral responses, including T cell activation status, the proportion of IgA-producing cells and circulating monocytes/neutrophils, revealed no evidence of spontaneous inflammation in CD74^ΔIEC^ mice (**Supplementary Figures 5B, C**).

However, a closer analysis revealed an increase in goblet cells accompanied by reduced ZO-1 expression in the intestinal epithelium (**Figures 2D–G**). Immunoblot analysis confirmed reduced ZO-1 protein expression in IECs from CD74 ^ΔIEC^ mice (**Figure 2G**). Immunohistochemical analysis revealed no apparent differences in the staining pattern or localization of Claudin-1, Claudin-2, Claudin-4, or Occludin between CD74 ^ΔIEC^ and WT mice (**Supplementary Figure 6**).

To determine whether the epithelial alterations observed in CD74 ^ΔIEC^ mice affected barrier function, we performed an FD-4 dextran assay and observed significantly higher plasma FD4 levels in CD74 ^ΔIEC^ mice than in WT controls (**Figure 2H**), suggesting a role for epithelial CD74 in maintaining intestinal barrier integrity. Because alterations in the gut microbiota can influence epithelial barrier integrity, we next asked whether IEC-specific deletion of CD74 altered the intestinal microbiota. 16S rRNA gene sequencing revealed no significant differences in overall microbial community composition between CD74 ^ΔIEC^ mice and WT control mice (**Supplementary Figure 7**).

To further assess whether this barrier defect rendered the epithelium more susceptible to stress, we subjected mice to radiation injury (RI). Following RI, while mortality was not increased, CD74 ^ΔIEC^ mice exhibited greater body weight loss compared to control mice (**Figure 2I**). Histological examination of the small intestine at day 3 after irradiation revealed that a greater proportion of CD74 ^ΔIEC^ mice displayed inflammatory cell infiltration and villus shortening compared with control mice (**Supplementary Figure 8**). Although the overall histological damage score did not reach statistical significance, CD74 ^ΔIEC^ mice showed a trend toward greater intestinal injury. Consistent with the weight loss data, levels of bacterial 16S rRNA in blood (26–28) were increased in irradiated CD74 ^ΔIEC^ mice compared with irradiated control mice (**Figure 2J**). Together, these findings indicate that loss of epithelial CD74 increases susceptibility to radiation-induced intestinal stress.

Together, these findings suggest that epithelial CD74 contributes to the maintenance of intestinal homeostasis and barrier integrity, as its loss is associated with reduced ZO-1 expression and increased intestinal permeability.

### CD74 deficiency results in increased proliferation in the intestinal epithelium

In addition to aforementioned leaky gut phenotype, we observed a modest increase in small intestinal length in CD74^ΔIEC^ mice compared with littermate WT mice (**Figure 3A**), suggesting altered epithelial turnover dynamics in CD74-deficient setting. Indeed, intestinal alkaline phosphatase (ALP) activity, a marker of enterocyte maturation, was reduced in the small intestine of CD74^ΔIEC^ mice (**Figure 3B**). It has been demonstrated that proliferation and differentiation are closely linked in the intestinal epithelium, and that increased proliferative activity within the crypt compartment can accelerate epithelial cell migration along the villus axis, potentially limiting full differentiation (3, 29, 30). To test whether CD74 regulates IEC proliferation, we examined IEC proliferation by Ki67 staining. The number of Ki67^+^ cells was significantly increased in the crypts of CD74^ΔIEC^ mice compared with control mice (**Figure 3C**), indicating enhanced crypt cell proliferation in the absence of CD74.

**Figure 3:**
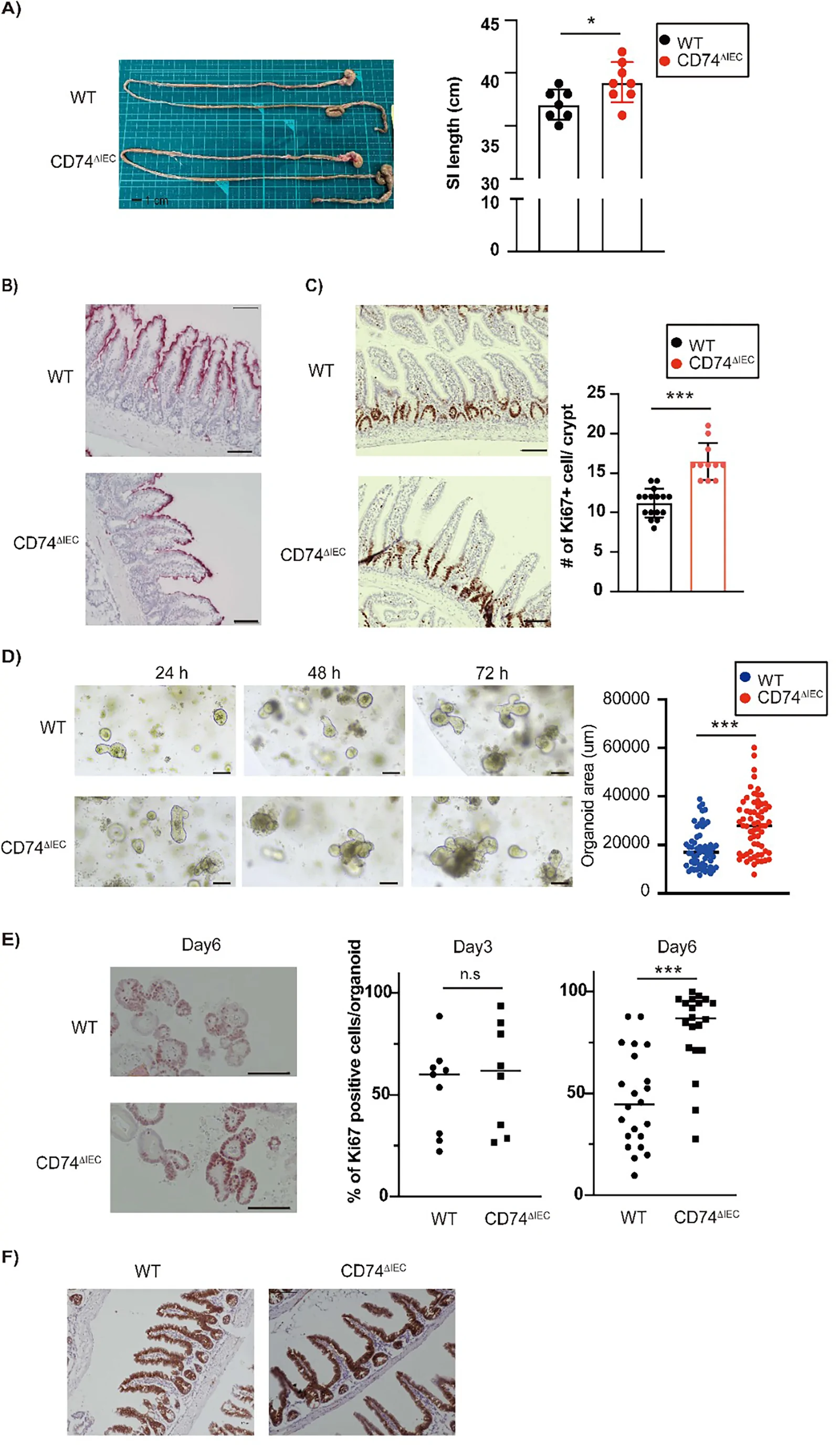
CD74 deficiency enhances the proliferative capacity of intestinal epithelial cells. **(A)** Gross morphology and quantification of small intestinal length in 3-month-old mice of CD74^ΔIEC^ mice and WT littermate controls. *p<0.05 **(B)** Representative ALP staining of distal small intestine from o CD74^ΔIEC^ mice and WT littermate controls. Scale bars, 100 µm. **(C)** Representative immunohistochemistry of distal small intestine for Ki67 and the numbers of proliferating crypt cells in CD74^ΔIEC^ mice and WT littermate controls (n = 3–5 crypts per mouse). Scale bars, 100 µm. ***p<0.001 **(D)** Representative phase-contrast images of small intestinal organoids derived from CD74^ΔIEC^ mice and WT littermate controls. Quantification of organoid area at day 4 is shown. Scale bars, 100 μm. *p<0.05. **(E)** Representative Ki67 immunostaining of day 6 small intestinal organoids derived from CD74^ΔIEC^ mice and WT littermate controls, with quantification of Ki67^+^ cells at days 3 and 6. Scale bars, 100 µm. ***p<0.001 **(F)** Representative immunohistochemical staining of β-catenin in the distal small intestine of CD74^ΔIEC^ mice and WT littermate controls. Scale bars, 100 μm.

To further assess the proliferative capacity of intestinal crypt cells, isolated crypts were cultured in Matrigel to generate organoids. Crypts from CD74^ΔIEC^ mice formed significantly larger organoids than those from WT mice (**Figure 3D**). Consistent with this finding, CD74-deficient organoids exhibited accelerated growth and reached larger sizes during culture. At day 3, CD74-deficient organoids already showed a modest increase in the proportion of Ki67^+^ cells compared with WT organoids, and this difference became more pronounced by day 6 (**Figure 3E**). Because canonical Wnt/β-catenin signaling is a key regulator of intestinal epithelial proliferation, we next examined β-catenin expression in the small intestine. Immunohistochemical analysis revealed comparable β-catenin staining patterns in WT and CD74^ΔIEC^ mice, with no obvious differences in expression or localization (**Figure 3F**). Taken together, these findings indicate that deletion of CD74 enhances epithelial proliferative capacity, as evidenced by increased crypt cell proliferation and larger organoids, without obvious changes in β-catenin immunostaining.

### CD74 deficiency is associated with alterations in mitochondrial state

To determine whether CD74 deficiency in IECs alters their transcriptional programs, we performed single-cell RNA sequencing on ileal IECs isolated from CD74^ΔIEC^ mice and WT littermate controls. Distinct epithelial cell clusters were identified based on the expression of representative marker genes, including enteroendocrine cells (EECs), enterocytes, goblet cells, intestinal stem cells (ISCs), Paneth cells (PCs), transit-amplifying cells (TAs), and tuft cells (**Figure 4A**). CD74 expression was enriched in ISCs and TA cells but was largely absent from PCs (**Figures 4A, B**), consistent with previous reports (17). Efficient deletion of CD74 in IECs from CD74^ΔIEC^ mice was confirmed. The overall distribution of epithelial cell subsets was comparable between WT and CD74^ΔIEC^ mice (**Figures 4C, D**), indicating that epithelial CD74 deficiency does not substantially alter epithelial cell composition under homeostatic conditions. Only modest transcriptional changes were observed in CD74-deficient IECs (**Figure 4E**), and a complete list of differentially expressed genes is provided in **Supplementary Table 1**. Nevertheless, gene set analysis identified enrichment of pathways related to mitochondrial organization, mitochondrial metabolism, and mitochondrial changes associated with apoptosis (**Figures 4F, G**). The genes contributing to these pathway analyses are listed in **Supplementary Table 2**. These findings suggest that the modest transcriptional changes associated with CD74 deficiency include alterations in mitochondrial-related pathways. TUNEL assays showed no difference in apoptosis between WT and CD74-deficient intestinal epithelial cells (**Supplementary Figure 9**), indicating that these pathway-level changes were not accompanied by an overt increase in epithelial cell death under steady-state conditions.

**Figure 4:**
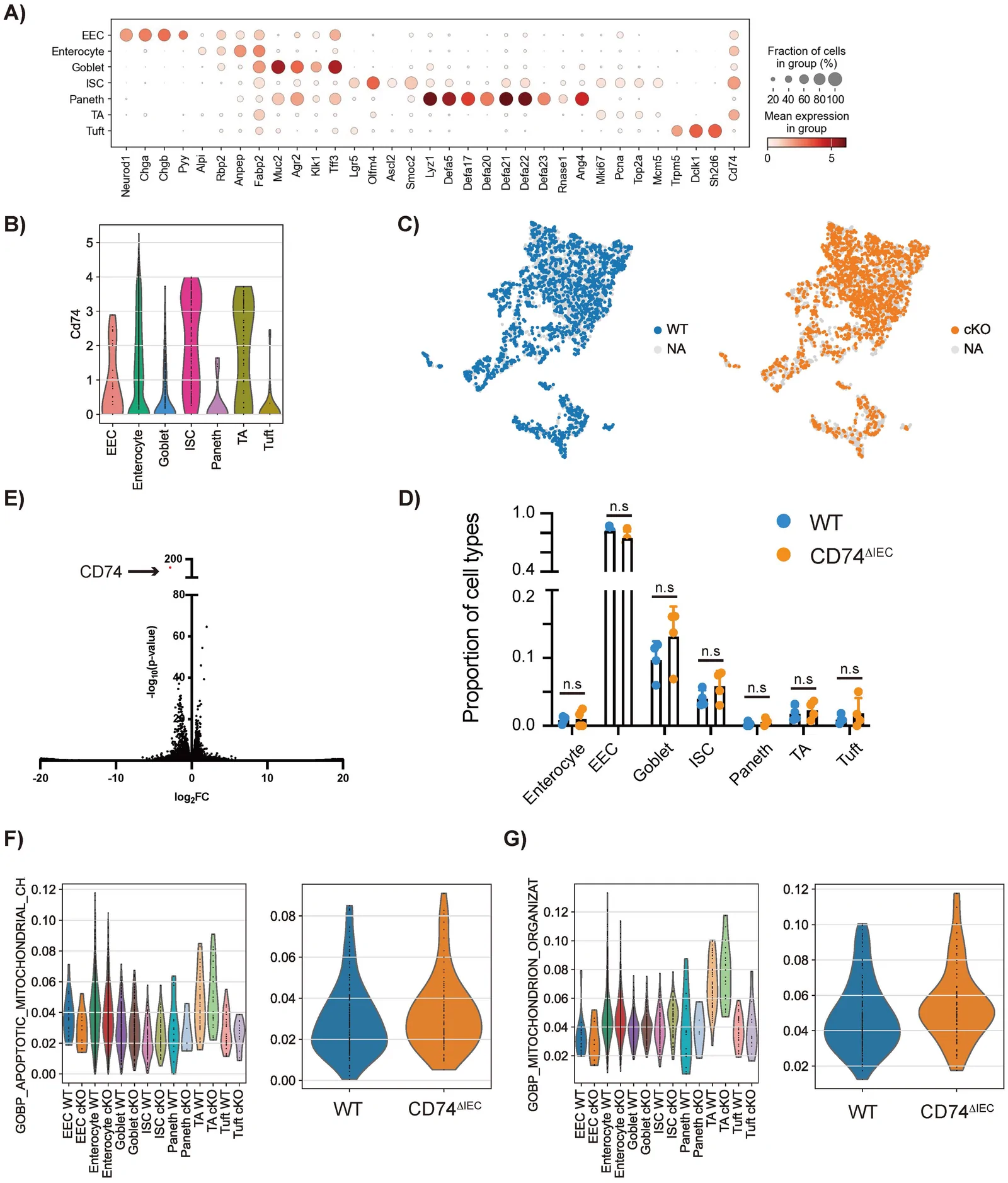
Single-cell RNA sequencing reveals preserved epithelial cell composition and enhanced mitochondrial programs in CD74^ΔIEC^ mice. Single-cell RNA sequencing of IECs isolated from pooled distal small intestinal tissues of three CD74^ΔIEC^ mice and three WT littermate controls (n = 3 per genotype). **(A)** Expression of canonical marker genes used to identify each epithelial cell population. **(B)** Violin plots depicting CD74 expression across the indicated epithelial cell lineages. **(C)** Uniform manifold approximation and projection (UMAP) visualization of single-cell RNA sequencing data from CD74^ΔIEC^ and WT mice. Cells from the indicated genotype are highlighted (WT, blue; CD74^ΔIEC^, orange), whereas cells from the other genotype are shown in gray (labeled as “NA”) for reference. **(D)** Relative abundance of epithelial cell subsets identified by single-cell RNA sequencing. The proportions of enteroendocrine cells (EECs), enterocytes, goblet cells, intestinal stem cells (ISCs), Paneth cells, transit-amplifying (TA) cells, and tuft cells are shown for CD74^ΔIEC^ and WT mice. **(E)** Volcano plot showing differentially expressed genes in WT versus CD74-deficient IECs. **(F, G)** Violin plots depicting pathway activity inference for mitochondrial-related pathways, including apoptotic mitochondrial changes **(F)** and mitochondrial organization **(G)**. The left panel shows all IEC subsets, whereas the right panel is restricted to TA and ISC populations.

To examine mitochondrial alterations, we performed ultrastructural analysis by transmission electron microscopy, which revealed an increased number of mitochondria in CD74-deficient IECs (**Figure 5A**). This observation was supported by MitoTracker Green FM staining, which demonstrated increased mitochondrial mass in CD74-deficient IECs (**Supplementary Figure 10**). Because mitochondrial characteristics can vary with epithelial differentiation status, we next examined Caco-2 cells, a human intestinal epithelial cell line that undergoes enterocyte-like differentiation during prolonged culture (31). Proliferating, immature Caco-2 cells cultured for 3 days exhibited higher mitochondrial mass and proliferation compared with differentiated cells cultured for 14 days (**Figures 5B, C**). These findings indicate that increased mitochondrial content is associated with a proliferative epithelial state. To determine whether mitochondrial regulation is linked to MIF–CD74 signaling, we used two complementary *in vitro* approaches. First, we examined Caco-2 cells, which constitutively produce high levels of MIF (32) and therefore maintain an autocrine MIF signaling loop. Pharmacological inhibition of MIF signaling with ISO-1 increased mitochondrial mass, accompanied by an increase in mitochondrial membrane potential as measured by MitoProbe TMRM, and enhanced epithelial proliferation (**Figures 5D, E**). We next examined primary intestinal organoids to evaluate the effects of exogenous MIF stimulation. Recombinant MIF reduced both mitochondrial mass and mitochondrial membrane potential, together with epithelial proliferation, in WT organoids (**Figures 5F, G**). Together, these findings indicate that modulation of MIF signaling is associated with changes in mitochondrial content, mitochondrial membrane potential, and epithelial proliferation. Whether these changes are directly mediated by CD74-dependent signaling, and how they relate mechanistically to the proliferative phenotype observed in CD74-deficient IECs, remains to be determined.

**Figure 5:**
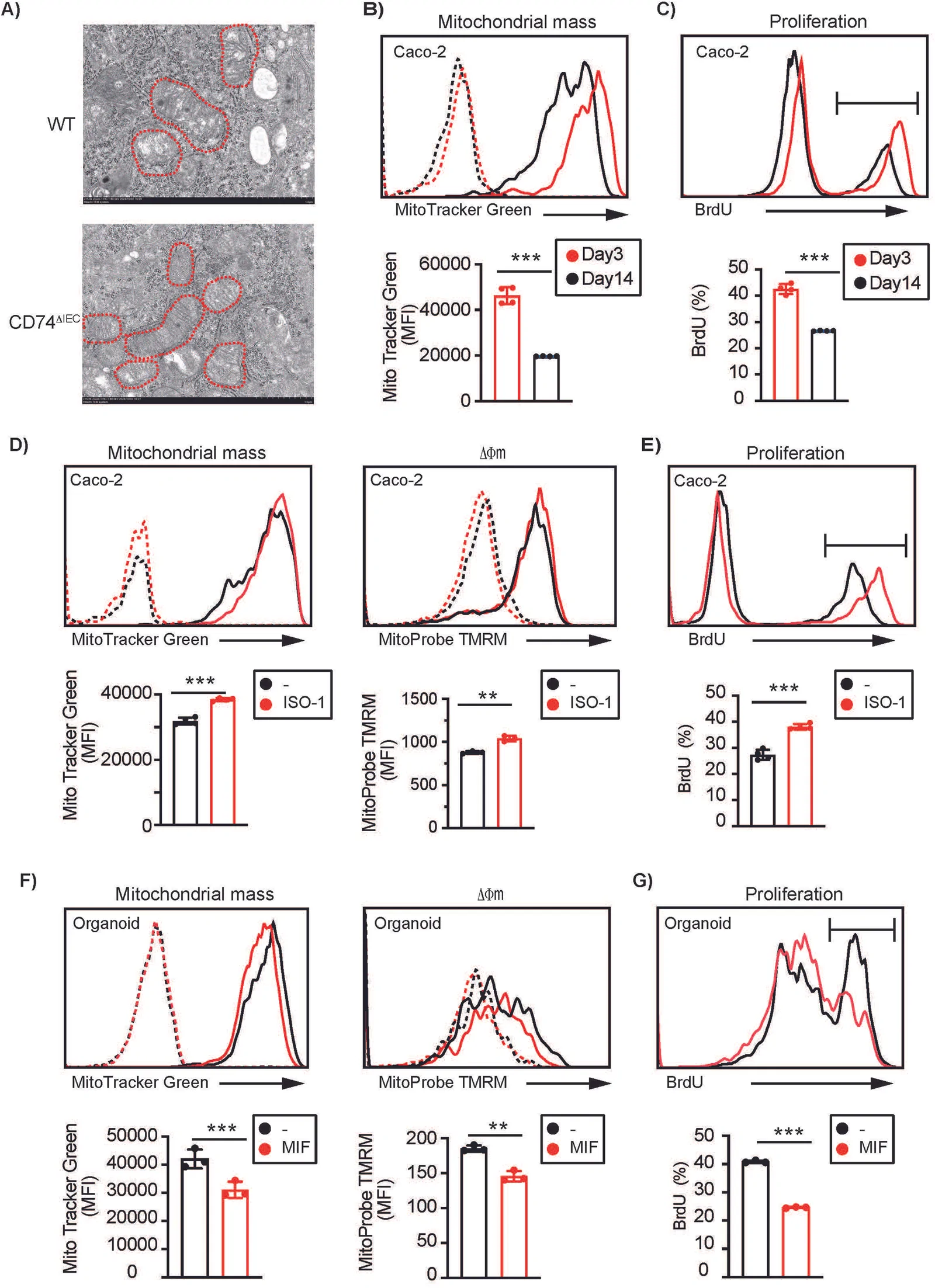
MIF signaling modulates mitochondrial parameters and epithelial proliferation. **(A)** Transmission electron microscopy of small intestinal epithelial cells showing ultrastructural mitochondrial changes. **(B)** MitoTracker staining of Caco-2 cells cultured for 3 or 14 days (n = 4 per group; two independent experiments), showing increased mitochondrial mass. ***p<0.001 **(C)** BrdU incorporation assay in Caco-2 cells cultured for 3 or 14 days, with BrdU (10 µM) added during the final 2 h of culture (n = 4 per group; two independent experiments). ***p<0.001 **(D)** Representative flow cytometric histograms and quantification of mitochondrial mass (MitoTracker) and mitochondrial membrane potential (MitoProbe TMRM) in Caco-2 cells cultured for 3 days with the MIF inhibitor ISO-1 (10 μg/ml) (n=4 per group; two independent experiments). ***p<0.001, **p<0.01. **(E)** BrdU incorporation assay of Caco-2 cells cultured for 3 days with ISO-1 (10 µg/ml), with BrdU (10 µM) added during the final 2 h of culture (n = 4 per group; two independent experiments). ***p<0.001 **(F)** Flow cytometric analysis of mitochondrial mass (MitoTracker) and mitochondrial membrane potential (MitoProbe TMRM) in small intestinal epithelial organoids derived from WT mice (n=3 per group; three independent experiments). ***p<0.001, **p<0.01. **(G)** BrdU incorporation assay of small intestinal epithelial organoids derived from CD74^ΔIEC^ and littermate WT mice. Organoids were treated with recombinant MIF, and BrdU (10 μM) was added during the final 4 h of culture (n = 3 per group; three independent experiments).

## Discussion

This study identifies epithelial CD74 as a cell-intrinsic regulator of small intestinal homeostasis. Although CD74^ΔIEC^ mice showed no gross morphological abnormalities under steady-state conditions, they exhibited increased intestinal permeability, a selective reduction of ZO-1 expression, enhanced crypt cell proliferation and organoid growth, and greater vulnerability to irradiation-induced stress. These findings reveal a previously unrecognized role for epithelial CD74 in supporting barrier function while limiting excessive epithelial expansion.

Previous studies of the intestinal epithelial MIF-CD74 pathway have primarily relied on fsystemic knockout models (15, 17). The increased intestinal permeability reported in MIF-deficient mice is consistent with the barrier defect observed in our CD74^ΔIEC^ mice (14), although these findings alone do not establish that the *in vivo* effects of MIF are mediated exclusively through epithelial CD74. By selectively deleting CD74 in IECs while preserving its expression in immune cells, our model distinguished epithelial-intrinsic functions of CD74 from the systemic immunological abnormalities associated with global CD74 deficiency.

Notably, the barrier defect in CD74^ΔIEC^ mice was associated with reduced ZO-1expression. In contrast, immunohistochemical analysis revealed no apparent differences in the staining patterns or localization of Claudin-1, Claudin-2, Claudin-4, or Occludin. Thus, our data provide quantitative evidence for reduced ZO-1 expression, whereas the observations for the other tight junction proteins remain qualitative. Moreover, 16S rRNA sequencing did not detect a significant shift in the overall microbial community structure of CD74^ΔIEC^ mice, indicating that CD74 deficiency was not associated with a major alteration in the overall composition of the intestinal microbiota.

Our findings further identify the intestinal microbiota and IFN-γ signaling as major regulators of epithelial CD74 expression. Broad-spectrum antibiotic treatment markedly reduced CD74 expression, whereas fecal microbiota transplantation restored it. In parallel, epithelial deletion of the IFN-γ signaling diminished epithelial CD74 expression, and recombinant IFN-γ directly induced CD74 expression in intestinal organoids. These results establish that epithelial CD74 is both microbiota-responsive and IFN-γ-inducible. However, they do not demonstrate that the microbiota regulates CD74 exclusively through IFN-γ. Moreover, the persistence of CD74 expression in the absence of epithelial IFN-γ signaling indicates that additional IFN-γ-independent pathways are involved. Previous studies have shown that germ-free or antibiotic-treated mice display increased intestinal permeability and reduced ZO-1 expression (33). These findings raise the possibility that microbiota-derived signals contribute to epithelial homeostasis partly through regulation of CD74 expression, although whether the barrier and proliferative phenotypes associated with CD74 deficiency are microbiota-dependent remains to be determined.

In addition to its effects on barrier function, CD74 deficiency increased crypt cell proliferation, small intestinal length, and organoid growth, while reducing intestinal alkaline phosphatase activity. Nevertheless, OLFM4^+^ stem cell and LYZ^+^ Paneth cell compartments were preserved, and scRNA-seq analysis revealed no major changes in the proportions of the principal epithelial cell populations. These results argue against gross disruption of epithelial composition or depletion of the stem-Paneth cell compartment. CD74^ΔIEC^ mice also exhibited an increase in MUC2^+^ goblet cells in the absence of overt baseline inflammation. This increase may represent a compensatory response to impaired barrier function, although its underlying mechanism remains unclear. Importantly, the increased number of Ki67^+^ crypt cells and the larger organoids do not establish that CD74 directly regulates ISC activity. CD74 may instead affect TA-cell proliferation, differentiation, survival, or other processes that influence overall organoid growth. Thus, the present data support a role for CD74 in limiting epithelial proliferative potential, whereas its specific function in ISCs remains to be determined. Larger single-cell datasets and lineage-resolved analyses will be required to identify subtle changes in epithelial cell states or differentiation trajectories.

Although individual transcriptional changes in CD74-deficient IECs were modest, gene set analysis identified coordinated alterations in pathways related to mitochondrial organization and metabolism. These transcriptomic findings should be regarded as supportive rather than definitive evidence of mitochondrial dysregulation. Consistent with the pathway analysis, CD74-deficient IECs contained increased numbers of mitochondria and showed increased mitochondrial mass. Functional perturbation of MIF signaling also altered mitochondrial potential *in vitro*: inhibition of MIF signaling with ISO-1 increased mitochondrial mass, membrane potential, and proliferation in Caco-2 cells, whereas exogeneous MIF reduced these parameters in WT intestinal organoids. These findings link MIF-sensitive signaling to epithelial mitochondrial state and proliferative activity. Whether such mitochondrial alterations directly cause the proliferative or barrier phenotypes observed in CD74^ΔIEC^ mice should be determined in future study.

We also obtained functional evidence that epithelial CD74 transduces MIF-induced signaling. MIF-induced ERK phosphorylation in Caco-2 cells was reduced by a CD74-blocking antibody, and MIF-induced ERK phosphorylation was detected in WT but not CD74-deficient intestinal organoids. These complementary results support a role for CD74 in MIF-induced ERK activation in intestinal epithelial cells. These findings are consistent with previous reports showing that MIF–CD74 signaling activates ERK in immune cells (10–13) and extend this observation to intestinal epithelial cells. However, these data do not establish that ERK directly mediates the increased proliferation or reduced ZO-1 expression caused by CD74 deficiency, suggesting that additional downstream pathways may be involved. Rescue or CD74 overexpression experiments were not performed, leaving the causal relationship unresolved. Similarly, the absence of an overt difference in β-catenin expression does not completely exclude a contribution of canonical Wnt signaling. The downstream pathways linking CD74 to epithelial proliferation, mitochondrial state, and ZO-1 expression therefore remain to be defined.

Taken together, our findings support a model in which microbiota-responsive and IFN-γ-inducible CD74 acts as an epithelial signaling receptor that contributes to barrier integrity and constrains epithelial proliferative and metabolic states. The principal advance of this study is the identification of epithelial CD74 as a cell-intrinsic regulator of intestinal homeostasis. Defining the signaling events that connect CD74 to ZO-1 expression, mitochondrial regulation, and epithelial proliferation will be an important direction for future investigation.

## Data Availability

The original contributions presented in the study are publicly available. This data can be found here: NCBI BioProject, accession number PRJDB45837 and NCBI GEO, accession number GSE351464.

## Supplementary material

Supplementary Figure 1 Microbiota and epithelial IFN-γ signaling contribute to CD74 expression in intestinal epithelial cells. **(A)** Representative flow cytometric histograms showing surface and intracellular CD74 expression on intestinal epithelial cells (IECs) isolated from the distal small intestine of untreated WT mice, mice treated with vancomycin or broad-spectrum antibiotic cocktail (AVNM), and Ifngr^ΔIEC^ mice **(B)** Quantification of the percentage of surface CD74-positive and intracellular CD74-positive IECs from the indicated groups. **(C)** Representative flow cytometric histograms showing surface and intracellular CD74 expression on distal small intestinal IECs from mice treated with a broad-spectrum antibiotic cocktail for 3 weeks, followed by fecal microbiota transplantation (or no treatment) for 7 days after antibiotic withdrawal. Mice were analyzed 7 days after the final fecal transfer. **(D)** Quantification of the percentage of cell-surface CD74-positive and intracellular CD74-positive IECs in mice after antibiotic cocktail treatment with or without fecal transfer.

Supplementary Figure 2 IFN-γ induces CD74 expression and CD74 contributes to MIF-induced ERK activation in intestinal epithelial cells. **(A)** Representative flow cytometric histograms showing surface CD74 expression on Organoid following stimulation with IFN-γ. Quantification of CD74 surface expression is shown on the right. **(B)** Representative flow cytometric histograms showing phosphorylated ERK (pERK) levels in Caco-2 cells stimulated with MIF for 20 min in the presence or absence of the CD74-blocking antibody LN2. Quantification of pERK-positive cells is shown on the right. **(C)** Representative flow cytometric histograms showing phosphorylated ERK (pERK) levels in Organoids stimulated with MIF for 20 min. Quantification of pERK-positive cells is shown on the right.

Supplementary Figure 3 CD74 and MHC II expression of CD74 ^ΔIEC^ mice. **(A)** Flow cytometric analysis of surface and intracellular CD74 expression on IECs from the distal small intestine and splenic B cells from CD74^ΔIEC^ mice and WT littermate controls. Representative plots are shown. (B-D) Flow cytometric analysis of surface MHC II expression on IECs from distal small intestine **(B)**, on dendritic cells **(C)** and B cell **(D)** from spleen or mesenteric lymph nodes (MLN) of CD74^ΔIEC^ mice and WT littermate controls. Representative plots are shown.

Supplementary Figure 4 H&E staining of the small intestine of CD74 ^ΔIEC^ mice. Representative H&E-stained sections of the small intestine from CD74^ΔIEC^ mice and WT littermate controls under steady-state conditions. Scale bars, 100 µm.

Supplementary Figure 5 Effects of epithelial CD74 deletion oVn immune cell populations. **(A)** Frequencies of naïve CD4^+^ T cells (CD4^+^ CD5^+^ CD62L^+^) and Tfh cells (CD4^+^ CD5^+^ PD-1^+^ CXCR5^+^) in spleen, Payer’s patches (PP), and mesenteric lymph nodes (MLN) of CD74^ΔIEC^ and WT control mice. **(B)** Frequencies of GC B cells (B220^+^CD5^-^GL7^+^CD38^-^) and IgA^+^ B cells (B220^+^IgA^+^) in spleen, Payer’s patches, and mesenteric lymph nodes of CD74^ΔIEC^ and WT control mice. **(C)** Frequencies of circulating monocytes (CD11b^+^Ly6C^high^) and neutrophils (CD11b^+^Ly6C^mid^) in blood of CD74^ΔIEC^ and WT control mice.

Supplementary Figure 6 Immunohistochemical staining of tight junction-associated proteins. Representative immunostaining of Claudin-1, Claudin-2, Claudin-4, and Occludin, in the distal small intestine of CD74^ΔIEC^ and WT littermate mice. Scale bars, 100 µ.

Supplementary Figure 7 β-diversity analysis of the fecal microbiota in CD74^ΔIEC^ and littermate WT mice. **(A)** Principal coordinates analysis (PCoA) based on Weighted UniFrac distances calculated from 16S rRNA gene sequencing of fecal samples from CD74^ΔIEC^ and WT control mice. Each point represents an individual mouse. **(B)** Distribution of pairwise Weighted UniFrac distances. Boxplots show the pairwise distances among WT mice (WT vs. WT) and between CD74^ΔIEC^ and WT control mice (CD74^ΔIEC^ vs. WT). No statistically significant differences in β-diversity were detected by PERMANOVA (p = 0.078) or ANOSIM (p = 0.231).

Supplementary Figure 8 Histological evaluation of intestinal injury following total body irradiation. **(A)** Representative hematoxylin and eosin (HE)-stained sections of the distal small intestine from CD74^ΔIEC^ and WT littermate mice at day 3 following total body irradiation (TBI). Scale bars, 100 μm. **(B)** Histological scoring of inflammation and tissue damage in the distal small intestine of CD74^ΔIEC^ and WT littermate mice at day 3 following TBI (n = 6 mice per group). Each symbol represents an individual mouse.

Supplementary Figure 9 Apoptosis in the small intestine of CD74^ΔIEC^ mice. TUNEL staining of SI sections from CD74^ΔIEC^ mice and WT littermate controls showing TUNEL^+^ apoptotic cells (green) with nuclear counterstaining (blue). A DNase-treated section was included as a positive control for the TUNEL assay. Scale bars, 100 µm.

Supplementary Figure 10 Analysis of mitochondrial mass in IECs from CD74^ΔIEC^ mice. Flow cytometry plots and quantification of MitoTracker mean fluorescence intensity (MFI) in ileal IECs from CD74^ΔIEC^ mice and control WT littermate controls (n = 3 per group; three independent experiments). ***p<0.001
